# REPATRIATION OF A PSYCHIATRIC PATIENT FROM BRAZIL TO CHINA

**DOI:** 10.1192/j.eurpsy.2023.1729

**Published:** 2023-07-19

**Authors:** A. S. Bragatto, V. M. Vourlis, J. Moesch, F. Timmermann, J. Brito, B. Reis

**Affiliations:** PSYCHIATRY, SANTA CASA DE MISERICÓRDIA DE SANTOS, SANTOS, Brazil

## Abstract

**Introduction:**

Developing or presenting with a serious mental illness whilst working offshore may result in substantial barriers to treatment, rehabilitation and repatriation to one’s home country, especially amid changes in border control practices over the COVID-19 pandemic. Further difficulties arise arise in relation to language and cultural barriers.

**Objectives:**

Our aim was to explore the experience of providing safe and effective treatment for a psychiatric patient from another country, culture and language using a non-medical interpreter.

**Methods:**

In this report, we describe the case of a 26-year-old Chinese citizen, a cargo ship crew member, who docked at the Port of Santos-Brazil in june 2021 with severe psychiatric disturbance.

**Results:**

Following the hospital assessment, the patient was admitted in the psychiatric ward and started the diagnostic research and treatment. After stabilization of the psychopathological condition, he was repatriated to his country of origin uneventfully. This case shows that treating and communicating with people who do not share the same language is challenging, in particular in a psychiatric context. The use of an interpreter is essential in the assessment process, but there are challenges in accessing and using these services.

**Conclusions:**

With a detailed multi-disciplinary rehabilitation plan a patient with serious mental illness can be rehabilitated in order to facilitate the repatriation in humanized ways and respecting all health protocols of COVID-19 pandemic.

**Disclosure of Interest:**

None Declared

